# Anticancer Effect of Pomegranate Peel Polyphenols against Cervical Cancer

**DOI:** 10.3390/antiox12010127

**Published:** 2023-01-05

**Authors:** Sandra Lucía Teniente, Adriana Carolina Flores-Gallegos, Sandra Cecilia Esparza-González, Lizeth Guadalupe Campos-Múzquiz, Sendar Daniel Nery-Flores, Raul Rodríguez-Herrera

**Affiliations:** 1 Food Research Department, School of Chemistry, Universidad Autónoma de Coahuila, Blvd. Venustiano Carranza and José Cárdenas, República Oriente, Saltillo 25280, Coahuila, Mexico; 2 School of Dentistry, Universidad Autónoma de Coahuila, Dra. Cuquita Cepeda de Dávila Ave, Adolfo López Mateos, Saltillo 25125, Coahuila, Mexico

**Keywords:** apoptosis, antiproliferation, anticancer, cell cycle arrest, cervical cancer, pomegranate peel, ROS

## Abstract

Polyphenols are a broad group of bioactive phytochemicals with powerful antioxidant, anti-inflammatory, immunomodulatory, and antiviral activities. Numerous studies have demonstrated that polyphenol extracts obtained from natural sources can be used for the prevention and treatment of cancer. Pomegranate peel extract is an excellent source of polyphenols, such as punicalagin, punicalin, ellagic acid, and caffeic acid, among others. These phenolic compounds have antineoplastic activity in in vitro models of cervical cancer through the regulation of cellular redox balance, induction of apoptosis, cell cycle arrest, and modulation of different signaling pathways. The current review summarizes recent data from scientific reports that address the anticancer activity of the predominant polyphenol compounds present in PPE and their different mechanisms of action in cervical cancer models.

## 1. Introduction

Worldwide, cervical cancer ranked fourth in incidence among the female population in 2018, with 570,000 cases and 311,000 deaths [1]. The main risk factors associated with the development of cervical cancer include HPV infection, age, tobacco use, use of oral contraceptives, and food intake [2]. Most cases of cervical cancer are caused by high-risk HPV infection causing DNA damage; however, recent studies have found that oxidative stress is also involved in this effect [3]. Oxidative stress (OS) refers to an imbalance between the production of reactive oxygen species (ROS) and the effectiveness of the antioxidant system to counteract the damage. This imbalance has been observed in cancer patients, even before the start of any treatment [4]. According to the National Cancer Institute, there are currently various treatments against malignant neoplasms, such as chemotherapy, radiotherapy, surgery for removal, immunotherapy, and hormonal therapy. However, not all patients are candidates for these treatments and it is usually necessary to resort to a combination of 2 or more therapies, which can have serious side effects on the patient’s body and immune system. Furthermore, these conventional therapies increase the generation of ROS, which can lead to toxic side effects in healthy tissues. This problem has led to physicians and researchers developing new possible treatments for this pathology. One of the most promising is the use of natural polyphenols for the development of anticancer drugs [4,5,6]. Dietary polyphenols are found mainly in foods of plant origin, such as fruits, vegetables, nuts, green tea, coffee, etc. [7]. The pomegranate (*Punica granatum* L.) is a widely consumed fruit belonging to the tree of the same name from the Lythraceae family. This berry is an excellent source of phytochemicals that have powerful antioxidant and anti-inflammatory effects [8]. The pomegranate peel (PE) is the inedible part of the fruit and is considered industrial waste. However, this by-product represents more than 40% of the total weight of the pomegranate and possesses numerous polyphenolic compounds at higher levels than those found in the juice and seeds [9,10,11]. Several studies have reported that polyphenols present in PPE have antiproliferative and antitumor activities in different cancer lines [12,13,14]. The aim of this review was to evaluate the molecular mechanisms involved in the anticancer effect of the polyphenolic compounds present in PP in different models of cervical cancer.

## 2. Methodology

To prepare this review, we conducted an electronic literature search using two databases: PubMed and Scopus. The search was performed using the following keywords: “Pomegranate peel,” “Phytochemical extraction,” “Polyphenol,” “Cervical cancer,” “Antioxidant,” and “Nanoparticles”. These keywords were used individually or in the following combinations: “Pomegranate peel + Polyphenol,” “Phytochemical extraction + Pomegranate peel,” “Pomegranate peel + Polyphenol + Cervical cancer,” and “Nanoparticles + Cervical cancer”. We included cell culture studies reporting the effects of pomegranate peel extract or its bioactive polyphenols. We excluded studies that were (1) only available in abstract form; (2) not written in English; and (3) books, meta-analyses, letters to the editor, and commentaries. We retrieved 427 articles, of which 92 were duplicates and were removed, and 225 articles were excluded for reasons 1, 2 and 3. Finally, 110 articles met the inclusion criteria and were included in this review.

## 3. Pomegranate Polyphenols

**Figure 1:**
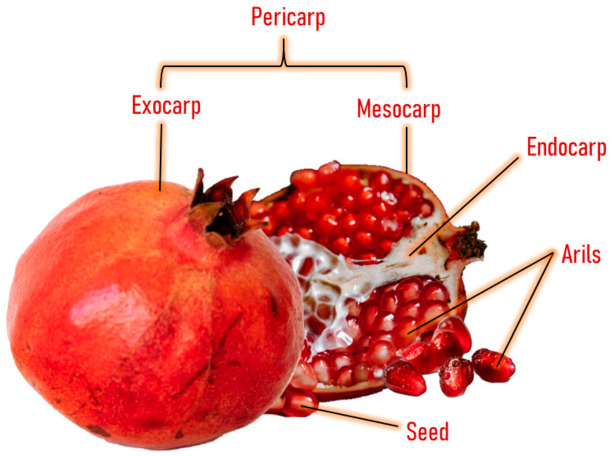
Anatomy of pomegranate fruit.

Pomegranate consists of light to dark red arils with little white seeds and a bright, deep red peel with an inner white tissue [15] (Figure 1). The inner part of the pomegranate fruit can be consumed as fresh fruit and juice, and both are widely consumed worldwide [16]. This fruit has been consumed since ancient times for its therapeutic and nutritional properties [17]. It possesses a high concentration of total polyphenols (83 mg/100 g of the edible portion or 250 mg/100 mL), with similar levels as red wine (203 mg/100 mL) and higher levels than green tea (103 mg/100 mL) [18]. The polyphenolic compounds present in this fruit include punicalagin, ellagic acid, punicalin, catechin, chlorogenic acid, gallic acid, epicatechin, caffeic acid, ferulic acid, delphinidin, and rutin [19,20,21].

**Figure 2:**
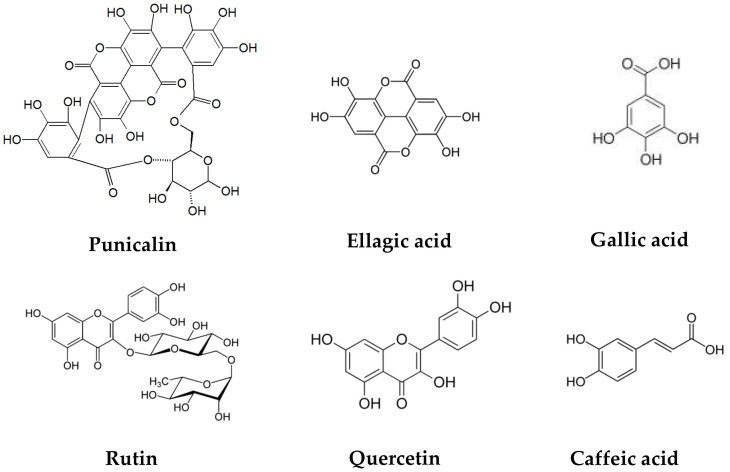
Chemical structures of polyphenols present in pomegranate peel.

The pomegranate peel has a significant amount of protein, polysaccharides, minerals (calcium, phosphorus, magnesium, potassium, sodium), and phenolic compounds such as flavonoids (catechin, epicatechin, quercetin, rutin, kaempferol, hesperidine, anthocyanins, procyanidins), hydrolyzable tannins (pedunculagin, punicalin, punicalagin), and phenolic acids (gallic, ellagic, vanillic, caffeic, ferulic, cinnamic, *p*-coumaric acids), among others [22,23,24]. (Figure 2).

## 4. Extraction and Characterization of Phytochemicals in Pomegranate Peel

For the correct standardization during the preparation of PPE, it is necessary to attend to different factors, including the solvent, pomegranate variety, extraction method, and use of green technologies [25]. The solvent choice has a significant influence on the concentration of polyphenols in PPE, as demonstrated by Al-Zoreky et al., who used extracts obtained with diethyl ether, 80% methanol (water-methanol), and distilled water to analyze the possible antimicrobial effect against various bacterial strains, finding that 80% methanolic extract had a much higher polyphenol content and exerted more potent antimicrobial activity [26]. Similarly, Tayel et al. tested different solvents, such as acetone, ethanol, methanol, ethyl acetate, and water, for the preparation and use of PPE as a possible antifungal agent, observing that methanolic extract had the best results, followed by ethanolic and aqueous extracts [27]. On the other hand, Venkataramanamma et al. standardized the extraction of PPE using a combination of water and ethanol, which are non-toxic food-grade solvents. The authors recommended this type of extraction at room temperature for 24–48 h at a 1:1 (*v*/*v*) concentration, considering it an efficient and eco-friendly option by minimizing the use of solvents and energy [28]. The variety and region of origin of the pomegranate fruit also influence the concentration and phenolic profile of PPE. When comparing the amount of ellagic acid obtained from the PPE of pomegranates from different regions, it was found that crops from Spain had 16.5 mg/g, Italy had 8.4 mg/g, and Egypt had 12.56 mg/g [29,30,31]. Ambigaipalan et al. identified numerous phenolic compounds in PPE from California crops using HPLC-DAD-ESI-MS. The compounds included hydrolyzable tannins (ellagic acid, ellagic derivatives, punicalagin isomers), phenolic acids (protocatechuic acid, vanillic acid, citric acid), anthocyanins (cyanidin-3-*O*-pentoside, delphinidin-3-*O*-glucoside, pelargonidin- 3-*O*-glucoside), and flavonoids (catechin, epicatechin, gallocatechin, quercetin, etc.) [32]. Meanwhile, using RP/HPLC/ESI/MS, Gonzalez-Castillo et al. found hydrolyzable (532.98 mg g^−1^) and condensed tannins (471.81 mg g^−1^), identifying punicalin, punicalagin, ellagic acid, and gallic acid as the main compounds of the PPE from Mexican pomegranate fruit [33].

There are different methods and analytical techniques for the extraction and identification of polyphenols from PP. Rajha et al. extracted polyphenols from PP using three different techniques: solid-liquid (SL), ultrasound (USE), and infrared extraction (IR). For all the techniques, deep eutectic solvents were used as eco-friendly alternatives to traditional solvents. High-performance liquid chromatography (HPLC) was used to identify and quantify the compounds, finding that the highest concentration was obtained using the IR technique [34]. In another study, Kaderides et al. compared the efficiency of microwave-assisted and ultrasound-assisted extraction of PP, finding that the microwave method was 1.7 times more efficient and only needed half the time compared to the ultrasound method. In addition, a high amount of ellagitannins was found, especially punicalagin (143.64 mg/g dry matter) [35]. García et al. used pressurized liquid extraction (PLE) with ethanol and water as an environmentally friendly alternative for the extraction of polyphenols from pomegranate peel, finding that this technique was effective for the extraction of total polyphenols but not for punicalagin recovery [36]. Other efficient green technologies for the extraction of polyphenols include enzyme-assisted solvent extraction (EASE) and enzyme-assisted supercritical fluid extraction (EASCFE). Mushtaq et al. analyzed both methods for the extraction of pomegranate peel, finding that EASCFE recovered twice as many polyphenols as EASE and increased their radical scavenging capacity. The authors used HPLC-DAD-ESI-MS to characterize the extracts, finding vanillic (108.36 µg/g), ferulic (75.19 µg/g), and syringic (88.24 µg/g) acids [37] (Table 1).

The use of polar solvents is effective in the recovery of polyphenolic compounds from plants [38]. Organic solvents such as diethyl ether [26], methanol [26,27], ethanol [27,28,36], acetone [27], and water [26,27,28] are suitable for the extraction of polyphenols from pomegranate peel. Aqueous acetone allows the extraction of high molecular weight flavonoids [27], while aqueous methanol recovers a greater quantity of total polyphenolic compounds [26,27]. The choice of extraction method (time, temperature, solvent ratio, and number of repeat extractions) depends on the chemical nature of the compounds to be recovered [38,39] and on the provenance of the pomegranate from which the peel is obtained [29,30,31,32,33].

## 5. Pomegranate Peel Extract for Cervical Cancer Treatment

Cervical cancer is a global burden, being the fourth most common cancer in women and responsible for 7.5% of female cancer-related deaths [40]. The primary cause of the development of cervical cancer is infection by human papillomavirus (HPV). There are 200 types of HPV; however, only 12 are related to carcinogenesis, with HPV-16 and HPV-18 being responsible for more than 60% of cases [41]. The integration of high-risk HPV DNA into the cellular genome leads to the expression of the *e6* and *e7* oncogenes, which interact with tumor suppressor factors and cell cycle regulators such as p53 and pRb, leading to deregulation and cell immortalization [42]. 

Currently, there are different procedures for the prevention and treatment of cervical cancer; the main ones are vaccination, radiotherapy, chemotherapy, and surgery. However, even with these treatments, patients in advanced stages have poor prognoses [43]. In chemotherapy, the most commonly used drugs are cisplatin, fluorouracil, and paclitaxel; however, they can produce severe side effects and drug resistance, causing tumor recurrence [44]. For this reason, the use of polyphenolic compounds has drawn attention as a viable alternative in the treatment of this pathology [45]. The pomegranate fruit and extracts obtained from its different parts possess high levels of polyphenol compounds that confer potent biological activities [46,47]. In recent years, pomegranate peel extract has stood out for its medicinal properties and biological activities. Fazio et al. analyzed the effect of acetone and methanolic PPE on the HeLa cervical cancer cell line. The cells were exposed to eight different concentrations (5, 15, 30, 60, 120, 240, 480, and 960 μg mL^−1^) of both extracts for a period of 72 h. Their results showed that both extracts had potent antiproliferative effects and marked scavenging activities in a dose-dependent manner. Furthermore, the methanolic extract activated caspases 3, 7, and 9, inducing DNA damage and triggering apoptosis. The total polyphenolic content of both extracts was measured, finding that the acetone extract contained 186 ± 8.7 mg GAE per g DW while the methanolic extract had 178.7 ± 2.5 mg GAE per g DW. Both extracts had similar phenolic content. The main compounds were punicalagin, pedunculagin, ellagic acid, gallic acid, and caffeic acid, among others. The authors determined that the polyphenols present in PPE were responsible for the antioxidant and anticancer activities in this cancer cell line [48].

Next, we will discuss the anticancer effect of the principal polyphenols present in PPE in cervical cancer models, individually or in combination with other compounds or drugs (Table 2).

## 6. Anticancer Effect of Pomegranate Peel Polyphenols on Cervical Cancer Models

Punicalagin (PCN), a hydrolyzable tannin present in pomegranate peel, provides numerous beneficial properties and is associated with anticancer activity in in vitro models because it intervenes in the cell cycle, proliferation/survival signals, and catabolic processes such as apoptosis and autophagy [49]. Zhang et al. observed that treatment with PCN at concentrations of 10 to 100 µM reduced the viability of the cervical cancer cell line ME-180 by up to 80%. The authors observed an increase in ROS generation and alterations in mitochondrial membrane potential, causing a cytotoxic effect on these cancer cells. Moreover, this treatment downregulated the expression of NF-kB protein and upregulated the expression of caspase-3 and -9, Bax, and p53 mRNA, thus stimulating apoptosis, which was corroborated by changes in the morphology of the cell’s nucleus [50]. Comparably, Tang et al. found that the viability of HeLa cells was affected when different concentrations of PCN were administered (12.5, 25, 50, 100, and 200 µM for 24, 36, and 48 h) in a time- and dose-dependent manner. This decrease in proliferation was due to the induction of cell cycle arrest in the G1 phase, caused by the downregulation of β-catenin and its downstream proteins cyclin D1 and c-myc, which are responsible for the transition from the G1 phase to the S phase. Additionally, treatment with PCN led to cell death by modulating the expression of apoptosis-associated proteins, downregulating the expression of anti-apoptotic Bcl-2, and upregulating the expression of pro-apoptotic Bax. Moreover, PCN stopped the progression of cell migration; this was demonstrated using the wound-healing assay, where treated cells did not recover in the same way as control cells. They also had higher expression of MMP-9 and MMP-2 proteins, which interfered with their invasive capacity [51]. A similar antiproliferative effect was observed by Xie et al. in SiHa and HeLa cells when different amounts of PCN were administered. The concentrations that showed the most significant antiproliferative effect were 40–67 μM, while higher concentrations (90–140 μM) caused total cell death. The results suggested that apoptosis induced by PCN treatment was due to the expression of caspases since *casp*3, *casp*7, and *casp*9 were upregulated. In addition, downregulation of *e6* and *e7* was observed, with an effect on their target STAT3 proteins and the Rb retinoblastoma protein [52]. 

STAT3 mediates the expression of genes involved in cellular proliferation and apoptosis processes. Mutations and structural changes have been detected in this protein in cancer cell lines [53]. Punicalagin has demonstrated a potent anticancer effect on different cervical cancer cell lines through multiple signaling pathways involved in cell death, including the downregulated expression of NF-kB protein [49,50]. NF-kB is a group of transcription factors that participate in processes of inflammation, viral replication, and the initiation and progression of cancer. Activation of nuclear factor kB, triggered by HPV infection, can inhibit cell death by stimulating the transcription of anti-apoptotic genes. NF-kB also upregulates the transcription of genes involved in proliferation, metastasis, and angiogenesis. We can determine that NF-kB works as part of a network. The cervical cancer microenvironment includes the expression of numerous genes and is related to multiple factors, including the response to polyphenols [54].

Another hydrolyzable tannin found mainly in pomegranate peel is punicalin (PUN), which demonstrates various therapeutic properties, including antioxidant, anti-inflammatory, hepatoprotective, antiviral, antibacterial, and anticancer activities [55]. Gonzalez-Castillo et al. observed that treatment with PUN in conjunction with ellagic acid (EA) (extracted and purified from pomegranate peel) had a dose-dependent cytotoxic and antiproliferative effect on HeLa cells. Furthermore, these authors observed that these polyphenols modulated the expression of apoptosis-associated proteins by inhibiting the activity of Bcl-2 and activating caspase 3. The authors also pointed out that these polyphenols inhibited the Akt/mTOR pathway by regulating the proteins involved in its activation [33]. In cervical cancer, PI3k/Akt/mTOR is generally dysregulated and, despite its role and regulatory mechanism not being completely elucidated, has the potential to be a biomarker for early diagnosis and possible therapeutic target for the treatment of this pathology [56,57]. Currently, evidence shows a synergistic effect in the combination of some polyphenolic compounds, presenting higher anticancer activity than when used individually. Some compounds only exhibit certain biological activities when they are combined with other polyphenols [58].

Gallic acid (GA) is a polyphenol found in the peel and arils of pomegranate. This compound has potent antioxidant, anti-inflammatory, antimutagenic, and anticancer activities and, in specific concentrations, can act as a pro-oxidant, causing apoptosis in various cancer cell lines [59,60,61,62]. Zhao & Hu detected the effects of GA on SiHa and HeLa cervical cell lines, reporting that cell viability was reduced in a dose-dependent manner using concentrations of 0, 10, 15, 20, 25, 30, and 40 µg/mL. The authors also analyzed the antiproliferative effect of this polyphenol using the BrdU assay, finding a significantly decreased percentage in both cell lines. In the HeLa cell line, proliferation was reduced 27% compared to the control group, to give 3.7%, and in the SiHa cell line, proliferation was reduced 29% compared to the control group, to give 3.3%. Additionally, GA reduced migration and invasion in both cell lines, demonstrated by the wound-scratch assay, inhibiting closure of the gap at concentrations of 10, 15, and 20 µg/mL. Further, these authors observed a decrease in the expression of ADAM 17, EGFR, and phosphorylated Akt (p-Akt) proteins, which are involved in cell motility and invasion, through different molecular mechanisms [63]. Park et al. analyzed redox state changes in GA-treated HeLa cells that inhibited growth and led to cell death. The authors observed a decrease in cell growth at 24 h of treatment, with an IC_50_ of 80 µM, and complete inhibition at a concentration of >100 µM by 72 h. Furthermore, GA was found to increase ROS levels in a time- and dose-dependent manner (50–400 µM, from a time phase of 30 min to 24 h), accompanied by the loss of mitochondrial membrane potential. Additionally, cellular levels of the antioxidant enzyme glutathione peroxidase (GSH) decreased when treatment was administered above 100 µM at 24 and 72 h [64]. Similarly, You et al. found that 80 µM GA reduced cell viability by 50% after 72 h of treatment. In addition, an increase in ROS was observed, accompanied by the loss of mitochondrial membrane potential. Finally, treatment with GA led to cell death [65]. Both scientific groups [64,65] noted an increase in ROS levels and loss of mitochondrial membrane potential. In healthy cells, polyphenols regularly interact with ROS to prevent cell viability from being affected [66]. However, some polyphenolic compounds can selectively target cancer cells and increase ROS levels, causing apoptosis and autophagy. Additionally, they can alter mitochondrial functions, such as oxidative phosphorylation, signaling pathways, and mitochondrial enzymes [67].

Another polyphenol compound found in PP is ellagic acid (EA) [68]. Li et al. observed that treatment with EA significantly decreased the viability of HeLa, SiHa, and C33A cervical cancer cell lines in a time- and dose-dependent manner (10, 20, and 30 µM; 24, 48, and 72 h). Additionally, EA had a dose-dependent apoptotic effect on HeLa cells caused by cell cycle arrest in the G1 phase via the regulation of STAT3 signaling and modulation of the expression of associated proteins (cyclins) [69]. Similar results were obtained by Narayanan et al. when the CaSki cell line was subjected to EA treatment for 48 h, in which cell cycle arrest occurred in the G1 phase; however, the authors attributed these results to an increase in the expression of both p21 mRNA and protein [70]. Gou et al. observed a decrease in the invasion capacity of HeLa cells when treated with different concentrations of EA (2.5, 5.0, and 10.0 µM), which was due to an increase in *igfb*7 (insulin-like growth factor-binding protein 7) gene expression causing the inhibition of the Akt/mTOR signaling pathway [71]. This pathway plays a role in cell growth, proliferation, differentiation, metabolism, and apoptosis; alterations in this pathway are involved in many pathologies, including cancer and tumor development. Kumar et al. analyzed the combination of EA with other phenolic compounds and found a synergistic effect with potential therapeutic utility. The authors detected an increase in the production of ROS and DNA damage, which led to cell death. Other effects produced by this combination were the restored activity of *p53* and *p21* genes and increased expression of the Bax proapoptotic protein. In addition, mRNA expression of the E6 oncoprotein of HPV significantly decreased in cervical cancer cells when different concentrations of a mixture of both polyphenols were administered, suggesting antiviral activity [72]. Polyphenols have proven to be active compounds against different viral infections through mechanisms involved in the growth cycle of viruses. These antiviral action mechanisms depend on the type of virus and whether a single polyphenol or polyphenolic extract is used. Several studies have indicated that this antiviral activity is due to inhibition of viral replication in the early stages of infection. More research is needed exploring the effect of polyphenolic compounds, individually or in groups, and their interaction with different viruses [70].

Caffeic acid (CA) is a phenolic compound found in pomegranate fruit, mainly in the peel and juice [73]. Different studies have reported its antioxidant, anti-inflammatory, antihypertensive, antidiabetic, and anticancer properties [74]. The antitumor activity of CA has been subject to in vitro and in vivo studies, demonstrating different molecular pathways through which this compound could be used as a treatment in different types of cancer, including cervical cancer [75]. Hsu et al. treated five cervical cancer cell lines with 50 µM CA, including HeLa, Siha, ME180, Caski, and pre-cancerous line Z172. These authors observed an increase in the expression of the E2F-1 transcription factor, which has an essential role in regulating the cell cycle, serving as a checkpoint, and inducing cell arrest in the S and G2/M phases [76]. In HeLa cell lines, treatment with CA (1–10 µM) for 24 to 48 h had a modulating effect on the expression of caspase-3; it also increased the expression of p53 and inhibited the activity of Bcl-2, which suppressed growth and induced cell death via the mitochondrial apoptotic pathway [77]. Ye et al. analyzed the antiproliferative and apoptotic effect of CA in conjunction with the antidiabetic drug metformin. A mixture of 100 µM CA with 10 mM metformin administered to the HTB-34 cell line, which has aggressive metastatic activity, caused an increase in intracellular oxidative stress that made the cell more susceptible to the metformin effect [78]. Metformin, a drug used in diabetes control, is also used to regulate epithelial-mesenchymal transition (EMT), transforming epithelial cells and conferring greater plasticity and ability to migrate. Metformin and CA treatment showed antimetastatic activity when administered individually in C-41 and SiHa lines; however, this activity was higher when administered together [79,80,81]. 

Chlorogenic acid (CLA) is a natural compound found in some plants and fruits, such as pomegranate. This polyphenol has several pharmacological effects, including antibacterial, antiviral, antioxidant, anti-inflammatory, and antimutagenic activities, and is listed by the China Food and Drug Administration (CFDA) as a potential anticancer drug [82,83,84]. Hemaiswarya & Doble analyzed CLA combined with other polyphenols, such as ferulic, caffeic, and *p*-coumaric acids, the phenolic derivative eugenol, and the antineoplastic drug 5-fluorouracil in HeLa cells. These authors observed that the apoptotic effect was more significant when the compounds were used together compared to when they were used individually, causing cell cycle arrest in the S and G2/M phases. Their findings indicated that the synergic effect was the result of the compounds having different target molecules involved in the cell cycle [85]. Similar results were obtained by Catanzaro et al. when they evaluated the activity of CLA combined with the anticancer drugs cisplatin and oxaliplatin in A431 and A431Pt cell lines. The results suggested that CLA bound to the anticancer drugs to form compounds with more significant activity against cancer cells. Therefore, as the authors suggested, CLA may help to combat drug resistance. However, more detailed studies are needed to determine possible interactions between different polyphenols and anticancer drugs [86]. 

Delphinidin (DPN) is an anthocyanidin found in the peel, arils, and juice of pomegranate [87]. Its anticancer activity has been observed in liver, breast, ovarian, lung, and cervical cancer cell lines [88,89,90,91]. Lazzé et al. analyzed the effect of DPN on cell proliferation, morphology, and induction of apoptosis in HeLa S3 cells. These authors observed that treatment with DPN at concentrations of 150 and 200 µM decreased cell viability to 59% and 50%, respectively. Moreover, the 24 h treatment showed a dose-dependent apoptotic effect, causing changes in morphology related to the cell death process, such as chromatin condensation and depletion of the nucleus. Finally, the mitochondrial membrane potential was analyzed, finding a 23.2% decrease in the number of treated cells compared to the control cells [92]. Tsuyuki et al. demonstrated that treatment with anthocyanidins, especially DPN, at concentrations of 25, 50, and 100 μM caused cytotoxicity and cell cycle abnormalities in HeLa and HeLa S3 cells. Additionally, DPN downregulated the expression of *c-Jun* (part of transcription factor AP-1) and induced the formation of autolysosomes and autophagosomes, leading to apoptosis [93]. AP-1 is a transcription factor that participates in cellular differentiation, proliferation, and apoptosis. The activity of this transcription factor can be induced by cytokines, viral infections, and physical/chemical stresses. In carcinogenesis, AP-1 has a crucial role in oncogenic transformation, and c-Jun protein positively regulates cell proliferation [94]. Therefore, as observed by Tsuyuki et al. [94], the inhibition of *c-Jun* expression in cervical cancer cells in parallel with the decrease in proliferation and cell cycle progression indicate that DPN may be a promising alternative for the treatment of this pathology.

Rutin (RU) is a polyphenol detected in the extract of pomegranate peel [95]. This compound has shown antioxidant, anti-inflammatory, antiangiogenic, proapoptotic, and antiproliferative activities, which could contribute to the treatment of different pathologies, such as cancer [96]. Khan et al. observed RU to possess a concentration-dependent (60–180 µM) pro-apoptotic effect on the Caski cell line. These authors demonstrated that RU modulated the expression of genes encoding the transcription factor Hes-1 and the transmembrane receptor Notch-1, the overexpression of which has been associated with cervical cancer progression. The results also showed an increase in ROS, alterations in Bax/Bcl-2 mRNA expression, decreased expression of CDK4 and cyclin D1, and an arrest of cell cycle progression in the G0/G1 phases [97]. Pandey et al. demonstrated that 24 h treatment of HeLa cells with RU significantly decreased cell viability in a dose-dependent manner (concentrations of 40, 80, 120, 160, and 200 μM), arrested the cell cycle at the G0/G1 phase, and reduced the expression of CDK4 and cyclinD1 mRNA. Moreover, these authors detected signals that indicated cell death, such as decreased mitochondrial membrane potential, induction of caspase-3, -8, and -9 activities, and the presence of apoptotic bodies. Additionally, RU treatment increased the expression of *p53* and *pRb* tumor suppressors and the *bax* gene and decreased the expression of E6 and E7 oncoproteins and the *bcl-2* gene [98]. Similar results were obtained by Pandey et al. in SiHa cells treated with RU (40–200 µM) for 24 h, in which cell death was induced in a dose-dependent manner. This apoptotic effect was associated with the upregulation of *bax* and *caspases* 3 and 9 and the downregulation of *bcl*-2 expression. Moreover, the authors demonstrated that RU downregulated the expression of the *jab1* oncogene, which plays a role in cancer progression by inactivating p53 and p27 tumor suppressors, leading to cell cycle arrest [99]. 

**Table 2:** Modulatory polyphenol impact on signaling pathways in cervical cancer cell lines.

Polyphenol	Concentration/ Time	Experimental Model	↑ Upregulation	↓ Downregulation	Final Effect	Reference
Punicalagin	10–200 μM 24 to 48 h	ME-180 HeLa SiHa	Bax Casp-3, 7, 9 STAT3 pRb	NF-KB Bcl-2 Cyclin D1 c-myc E6 and E7	Inhibition of cell proliferation and induction of apoptosis Cell cycle arrest	[50,51,52]
Punicalin	100, 500, and 1000 ppm 24, 48, and 72 h	HeLa	Casp-3	Bcl-2	Suppression of cell viability Induction of apoptosis	[33]
Gallic acid	10–400 μg/mL 30 min to 72 h	HTB-35 HeLa	ROS	ADAM17 EGFR p-Akt GSH	Cell cycle arrest Induction of apoptosis/ necrosis Inhibition of cell migration	[63,64,65]
Ellagic acid	2.5–30 μM 24, 48, and 72 h	HeLa SiHa C33A	STAT3 IGFB7 ROS P53 P21 Bax	Cyclin E6	Inhibition of cell proliferation and induction of apoptosis Cell cycle arrest at G1 phase Suppression of Akt/mTOR pathway Induce DNA damage	[69,70,71,72]
Caffeic acid	1–100 μM 24 to 48 h	HeLa SiHa ME180 Caski Z172	E2F-1 Casp-3 p53	Bcl-2	Cell cycle arrest at S and G2/M phases	[76,77,78]
Chlorogenic acid	IC_50_ ~ 10(-4) M 24 h	A431 A431Pt	___	___	Binds with cisplatin and oxaliplatin to oppose drug resistance	[85,86]
Delphinidin	25–200 μM 24 h	HeLa HeLa S3	___	c-Jun	Induces autophagosome formation Inhibition of cell proliferation and induction of apoptosis	[93,94]
Rutin	40–200 μM 24 h	Caski SiHA	ROS Bax p53 pRb Casp-2, 8 and 9 p27	Hes-1 Notch-1 Bcl-2 CDK4 Cyclin D1 E6 and E7 Jab1	Cell cycle arrest at G0/G1 phase Suppression of cell viability Induction of apoptosis	[97,98,99]

**Abbreviations:** Bax (Bcl-2-associated X protein); Casp (caspase); E2F-1 (E2F Transcription Factor 1); IGFBP7 (Insulin Like Growth Factor Binding Protein 7); ROS (Reactive Oxygen Species); STAT3 (Signal transducer and activator of transcription 3); ADAM17 (ADAM Metallopeptidase Domain 17); Atgs (autophagy-related protein); Beclin-1 (B-cell lymphoma 2); c-myc (Myc proto-oncogene); CDK4 (Cyclin-dependent kinase 4); c-Jun (Jun Proto-Oncogene); EGFR (Epidermal growth factor receptor); Hes-1 (hairy and enhancer of split-1); Jab-1 (Jun activation domain-binding protein 1); Mcl-2 (Induced myeloid leukemia cell differentiation protein); NF-KB (nuclear factor kappa-light-chain-enhancer of activated B cells); p-Akt (phosphorylated Akt); PI3K (Phosphoinositide 3-kinases); GSH (glutathione).

## 7. Bioavailability Limitations and the Use of Nanotechnology

Notwithstanding the evidence provided by numerous studies that have demonstrated the effect of polyphenols in cancer treatment, the issue that remains unresolved is the bioavailability of these compounds in vivo. This is because polyphenols have to interact with the food matrix, gut microbiota, and metabolic processes involved with digestion and absorption. For this reason, nanotechnology has been suggested to protect them from degradation and enable higher concentrations to reach target cells [100].

Nanotechnology has gained importance in recent years in cancer treatment and diagnosis. In the treatment with polyphenols, nanoparticles act as vehicles that allow the chemical compounds to be transported more efficiently by crossing biological barriers more easily, thus increasing their efficacy and reducing side effects. Different nanomaterials are used as transport systems through the body, such as micelles, liposomes, dendrimers, nanoemulsions, etc. These materials enable compounds to reach target tumor cells with a high level of specificity, thereby reducing collateral damage to healthy cells [101,102].

Currently, nanostructures with different sizes, shapes, and materials, such as proteins, polysaccharides, lipids, synthetic polymers, and inorganic materials, have been developed. However, nanoparticles with structures made from natural materials have better biological and physicochemical properties than those made from synthetic materials. Natural nanoparticles have higher biocompatibility, are biodegradable, and do not generate immune responses [103,104].

PPE has been used in the synthesis of gold, silver, copper, iron, and zinc oxide nanoparticles, among others, since they can act as reducing agents and stabilizers and are eco-friendly alternatives to toxic chemicals that pollute the environment. These functional PP nanoparticles can help in the development of more efficient therapeutics, as they are less cytotoxic, more effective, and have better bioavailability [105,106,107,108,109]. Khan et al. synthesized silver nanoparticles using PP extract and analyzed their effect on cancer cell lines. The authors observed a dose-dependent decrease in cell viability, the accumulation of ROS, morphological changes, and DNA fragmentation, suggesting anticancer potential [110]. Studies using functional nanoparticles with PP polyphenols in cervical cancer models are needed.

## 8. Future Prospects and Conclusions

According to the information obtained in the present study, pomegranate peel polyphenols seem to be a viable alternative for the prevention and treatment of cervical cancer. Recent studies have demonstrated that PP polyphenols, independently or in combination with other compounds or drugs, possess antiproliferative activities in cervical cancer models through the induction of apoptosis, cell cycle arrest, inhibition of DNA synthesis, and modulation of different signaling pathways. To improve bioavailability in more complex models, various research results reported in this paper suggest multiple alternatives, such as using two or more polyphenols together to achieve a synergistic effect, the encapsulation of polyphenolic extracts, and the use of nanotechnology. Functional nanoparticles possess strong anticancer activity, low toxicity, increased accumulation within the tumor, and faster kidney clearance. Being able to modify the surface of these nanoparticles and adding polyphenolic compounds makes them the perfect candidate for cancer treatment. In cervical cancer, the use of nanoparticles through the vaginal route would allow drug administration directly to the cervix, improving its action and reducing patient side effects. Therefore, the development of functional nanoparticles with biocompatible materials is expected in the near future, along with the combination of different polyphenols to replace current drugs in the treatment of cancer. Thus, the technology behind functional nanoparticles needs further study in order to be used safely in humans.

## Figures and Tables

**Table 1:** Extraction technologies and analytical techniques used to obtain and characterize polyphenol compounds from pomegranate peel.

Technology	Extraction Conditions	Identification Method	Outcome	Ref.
SL	Diethyl ether; 80% methanol; water 1 h Room temperature	Not indicated	DE: 6.2 mg GAE/g MW: 262.5 mg GAE/g W: 82.5 mg GAE/g	[26]
SL	Water/ethanol 1:1 Room temperature 24–48 h	RP-HPLC	Identification of punicalagin A and B, ellagic acid	[28]
USE	Acetone (70%) 20 min at 30 °C	HPLC-DAD-ESI-MS	Identification of seventy-nine phenolic compounds	[32]
SL	Water 60 °C for 30 min	RP/HPLC/ESI/ MS	Hydrolyzable tannins: 532.98 mg g^−1^ Condensed tannins: 471.81 mg g^−1^	[33]
SL USE IR	Deep eutectic solvents 50 °C for 90 min	HPLC	SL: 13 mg/g DM US: 114 mg/g DM IR: 152 mg/g DM	[34]
MAE USE	MAE: 50% aqueous ethanol LSR: 60:1 mL/g 600 W for 4 min UAE: water LSR: 32.2:1 mL/g Amplitude level: 39.8% Pulse: 1.2/1 at 34.7 °C for 10 min	HPLC-UV–vis	Punicalagin MAE: 143.64 mg/g DM USE: 138.8 mg/g DM	[35]
PLE	Ethanol (77%) 200 °C Pressure 1500 psi for 20 min	HPLC-DAD-ESI-TOF/MS	TPC: 164.3 ± 10.7 mg GAE/g DM Punicalagin: 17 ± 3.6 mg/g DM	[36]
EASEEASCFE	CK: cellulase, pectinase, and protease; (50:25:25) at 3.8%, 49 °C pH 6.7 for 85 min	HPLC-DAD-ESI–MS	TPC: 301.53 mg GAE/g Identification of gallic, caffeic, *p*-coumaric, ferulic, syringic, sinapic, and vanillic acids	[37]

**Abbreviations:** SL: solid-liquid extraction, USE: ultrasound-assisted extraction, IR: infrared extraction, MAE: microwave-assisted extraction, PLE: pressurized liquid extraction, EASE: enzyme-assisted solvent extraction, EASCFE: enzyme-assisted supercritical fluid extraction, CK: cocktail enzyme, DM: diethyl ether, MW: water/methanol, W: water, TPC: total polyphenol content, GAE: mg of gallic acid equivalent, DM: dry matter.
